# Investigation of the implementation and utilization of ketogenic diet therapy in China: For the Commission on Standardized Development of Epilepsy Centers and Commission on Ketogenic Diet, China Association Against Epilepsy

**DOI:** 10.1186/s42494-026-00242-w

**Published:** 2026-04-01

**Authors:** Wenjie Xi, Yicong Lin, Shujuan Tian, Jianxiang Liao, Yuping Wang

**Affiliations:** 1 Department of Neurology, the First Hospital of Hebei Medical University, Hebei, Shijiazhuang, 050051 China; 2 Department of Neurology, Xuanwu Hospital, Capital Medical University, No. 45 Changchun Street, Xicheng District, Beijing, 100053 China; 3 Neuromedical Technology Innovation Center of Hebei Province, Shijiazhuang, 050000 China; 4 Department of Neurology, Shenzhen Children’s Hospital, Shenzhen, 518000 China; 5 Department of Neurology, Handan Central Hospital, Handan, Hebei 056001 China; 6 Center of Epilepsy, Beijing Institute for Brain Disorders, Capital Medical University, Beijing, 100053 China; 7 Center for Sleep and Consciousness Disorders, Beijing Institute for Brain Disorders, Beijing, 100053 China; 8 Collaborative Innovation Center for Brain Disorders, Capital Medical University, Beijing, 100053 China; 9 Hebei Hospital of Xuanwu Hospital, Capital Medical University, Shijiazhuang, 050000 China; 10 Beijing Key Laboratory of Neuromodulation, Beijing, 100053 China

**Keywords:** Ketogenic diet therapy, Epilepsy, Epilepsy centers, Questionnaire

## Abstract

**Background:**

Ketogenic diet therapy (KDT), characterized by a high-fat, low-carbohydrate composition with adequate protein and other nutrients for growth, has emerged as an effective therapeutic approach for drug-resistant epilepsy. This study was designed to investigate the existing challenges in KDT implementation and evaluate its current utilization status within the Chinese healthcare context.

**Methods:**

From August 27 to September 5, 2024, a comprehensive questionnaire was disseminated to 418 epilepsy centers throughout China via the Commission on Standardized Development of Epilepsy Centers and the Commission on KDT, which operate under the auspices of the China Association Against Epilepsy (CAAE). The distribution leveraged both email and WeChat platforms to ensure broad reach and accessibility. The survey instrument encompassed five key domains: (1) baseline characteristics of surveyed epilepsy centers, (2) clinical application of KDT, (3) the efficacy of KDT: perspectives from epilepsy centers, (4) the adverse effects of KDT, and (5) implementation challenges and recommendations for KDT adoption. All the collected data were analyzed using descriptive statistical methods.

**Results:**

We received 258 completed questionnaires, for a response rate of 62%. Among the responding epilepsy centers, the classic KDT (cKDT) was utilized by 63% of the centers, the modified Atkins diet (MAD) was utilized by 40% of the centers, the low-glycemic-index treatment (LGIT) was utilized by 20% of the centers, and the medium-chain triglyceride (MCT) diet was utilized by 25% of the centers. The most significant barrier to KDT implementation was identified as limited KDT-related expertise among clinical practitioners, reported by 83% of the respondents. Additionally, improving patient and family education programs on KDT management was the most frequently cited factor (93%) for increasing KDT utilization. The data revealed strong clinical need for promoting KDT implementation, particularly in the management of super-refractory status epilepticus and refractory status epilepticus. Among the 120 epilepsy centers that implemented KDT, 27 reported cases of gastrointestinal adverse reactions.

**Conclusions:**

This study constitutes the first nationwide comprehensive investigation into the clinical application of KDT in China. The results reveal a notably limited adoption of KDT across epilepsy centers, despite its well-documented therapeutic efficacy and favorable safety profile. The current implementation remains limited to a select number of specialized centers and a relatively small patient population, primarily due to insufficient awareness and understanding among healthcare practitioners. These findings provide evidence-based insights that underscore the necessity for strategic initiatives to promote wider implementation of KDT in clinical practice.

**Supplementary Information:**

The online version contains supplementary material available at 10.1186/s42494-026-00242-w.

## Background

Epilepsy, which is among the most prevalent and debilitating neurological disorders, is primarily managed through antiseizure medication. While the majority of patients achieve seizure freedom with antiseizure medications, a substantial proportion (30%−40%) develop drug-resistant epilepsy (DRE). The therapeutic arsenal for DRE encompasses surgical interventions, neurostimulation techniques, and ketogenic diet therapy (KDT), which is a well-established alternative for patients who are refractory to pharmacological treatment and ineligible for surgical procedures. The KDT, characterized by its high-fat, low-carbohydrate food composition with adequate protein and other nutrients for growth, induces metabolic alterations similar to those observed during fasting states [1, 2]. This dietary intervention promotes the production of ketone bodies, which serve as alternative energy substrates for the brain, displacing glucose and demonstrating both antiseizure and antiepileptogenic effects [3]. Currently, the therapeutic landscape of KDT includes the classical KDT (cKDT) and its modified versions: the modified Atkins diet (MAD), low-glycemic-index treatment (LGIT), and medium-chain triglyceride (MCT) diet, all of which have demonstrated efficacy in DRE management [4–6]. The growing scientific interest in KDT stems from its demonstrated clinical effectiveness and favorable safety profile, positioning it as a promising therapeutic modality in epilepsy treatment.

The therapeutic potential of KDT in DRE management has been substantiated by numerous evidence-based studies across diverse patient populations. Extensive research from China, encompassing both prospective and retrospective clinical investigations, has consistently demonstrated the high feasibility, safety profile, and therapeutic efficacy of KDT in pediatric populations [7–9]. Notably, two of these studies specifically addressed the application of KDT in infants under 2 years of age, expanding the evidence base for this vulnerable demographic [7, 9]. The therapeutic scope of KDT has progressively extended to adult epilepsy management, supported by emerging clinical evidence of their feasibility and tolerability in this population. A longitudinal observational study conducted in Colombia reported significant clinical improvements in adult patients following KDT intervention [10]. Furthermore, a prospective observational study revealed that the KDT demonstrated moderate efficacy as an adjunctive therapy for adult DRE patients [11]. The therapeutic benefits of KDT were further corroborated by a randomized controlled trial involving 30 DRE patients [12]. The accumulating body of evidence has solidified the position of the KDT as an established therapeutic modality for epilepsy, with its clinical indications continuing to expand. Current research trends suggest a growing acceptance of KDT across various age groups and epilepsy subtypes, supported by their demonstrated efficacy and manageable adverse effect profile.

KDT has been associated with a spectrum of generally mild and manageable adverse effects, primarily gastrointestinal disturbances, metabolic alterations (hyperketonemia and hyperlipidemia), and potential nutritional imbalances (hypoglycemia and hypoproteinemia), with rare occurrences of kidney stones. While longitudinal studies have identified gastrointestinal symptoms and hyperlipidemia as the most prevalent adverse effects of sustained KDT [13, 14], the overall safety profile remains favorable when this therapy is implemented under proper medical supervision. The transient nature and clinical management of these effects, coupled with appropriate monitoring protocols, support the continued use of KDT as a safe therapeutic intervention for eligible patients.

In summary, KDT has been established as an effective and safe therapeutic intervention for epilepsy. However, international studies have identified significant barriers to its widespread implementation. In the United Kingdom, research has highlighted two primary obstacles: insufficient referral rates and limited funding availability [15, 16]. North American studies have identified different challenges, particularly the shortage of qualified dietitians and inadequate parental understanding of the dietary protocol [17]. The American context reveals additional barriers, including resource limitations, insufficient interdisciplinary collaboration, and the absence of standardized clinical guidelines [3]. French pediatric neurologists have reported distinct concerns, emphasizing palatability issues and the perceived restrictiveness of the cKDT protocol [18]. Within the Chinese healthcare context, 2016 data indicated that 34.1% of epilepsy centers administered KDT to a total of 4,737 patients [19]. While China has made significant progress in standardizing the development of epilepsy centers over the past decade, particularly in terms of pharmacotherapy, surgical interventions, and neuromodulation therapies, the current status of KDT implementation remains unclear. Critical questions persist regarding existing clinical application patterns and potential developmental barriers that may require intervention. Notably, comprehensive investigations into these aspects have not yet been conducted in the Chinese context.

This research gap presents a valuable opportunity to generate evidence-based insights that could inform the China Association Against Epilepsy's (CAAE) strategic initiatives to promote KDT adoption and development. The present survey was designed to achieve two primary objectives: (1) to investigate current KDT implementation practices across Chinese epilepsy centers, and (2) to identify and characterize the specific barriers hindering its broader application in clinical practice.

## Materials and methods

A comprehensive survey was distributed to 418 epilepsy centers accredited by the Commission on Standardized Development of Epilepsy Centers and the Commission on KDT under the CAAE. This study employed a structured questionnaire consisting of 30 items (detailed in the Supplementary Information), which were systematically organized into five key domains: (1) baseline characteristics of surveyed epilepsy centers, (2) clinical application of KDT, (3) the efficacy of KDT: perspectives from epilepsy centers, (4) the adverse effects of KDT, and (5) implementation challenges and recommendations for KDT adoption. The survey period for this questionnaire spanned from January 1, 2023, to December 31, 2023. The questionnaire was distributed through both email and WeChat platforms to epilepsy centers in China from August 27 to September 5 2024. Each participating center was granted a single submission opportunity, with the survey closing definitively on September 10, 2024.

The study design incorporated several quality control measures: (1) mandatory institutional-level responses rather than individual practitioner perspectives, (2) anonymous data collection to ensure respondent confidentiality, and (3) explicit requirements for data accuracy and truthfulness. The collected data were analyzed using descriptive statistical methods, with calculations performed for each variable to ensure comprehensive data representation. We also used the chi-square test to compare whether there were differences in the implementation of ketogenic diets among epilepsy centers of different tiers.

## Results

A total of 258 questionnaires were completed, with an overall response rate of 62%. The responding centers included 103 primary, 129 secondary, and 26 tertiary epilepsy centers. All the statistical data are presented as absolute numbers accompanied by corresponding percentages.

### Baseline characteristics of surveyed epilepsy centers

China's epilepsy diagnosis and treatment system comprises a three-tier structure, consisting of 165 primary epilepsy centers, 214 secondary epilepsy centers, and 39 tertiary epilepsy centers. The response rates for primary, secondary, and tertiary centers were 62% (103/165), 60% (129/214), and 67% (26/39) respectively. The distribution of the types of epilepsy centers was as follows: 24% (62/258) were pediatric-only, 29% (75/258) were adult-only, and 47% (121/258) were adults and children.

### Clinical application of KDT

KDT were implemented in 47% (120/258) of the surveyed epilepsy centers and were distributed across different tiers: 19% (20/103) were primary centers, 59% (76/129) were secondary centers, and 92% (24/26) were tertiary centers. The adoption rates of KDT exhibited statistically significant variations across primary, secondary, and tertiary epilepsy centers (*χ*^2^ = 47.56, *P* < 0.001). The post hoc analysis with Bonferroni correction revealed statistically significant differences in ketogenic diet implementation rates across all pairwise comparisons of epilepsy center tiers. Specifically, significant disparities were observed between primary and secondary centers (adjusted *P* < 0.0167), primary and tertiary centers (adjusted *P* < 0.0167), as well as between secondary and tertiary centers (adjusted *P* < 0.0167) (see Table 1). Analysis of patient demographics revealed that 68% (81/120) of these centers exclusively administered KDT to pediatric patients (aged ≤ 18 years), with distributions across 90% (18/20) primary, 67% (51/76) secondary, and 50% (12/24) tertiary centers. Adult-exclusive KDT implementation was reported by 7% (9/120) of centers, comprising 5% (1/20) of primary centers and 11% (8/76) of secondary centers. A total of 25% (30/120) of the centers provided KDT to both pediatric and adult populations; 5% (1/20) of the centers were primary centers, 22% (17/76) were secondary centers, and 50% (12/24) were tertiary centers (see Table 2).

**Table 1:** The Comparison of KDT Adoption Rates Across Different Tiers of Epilepsy Centers

	Adoption	Non-adoption	Adoption rates (%)	*χ*^2^	*P*
Primary centers	20	145	12%	26.96	< 0.001*
Secondary centers	76	138	36%	45.54	< 0.001#
Tertiary centers	24	15	62%	9.35	0.002**

The numbers in the table represent the number of epilepsy centers, whereas the percentages in parentheses indicate their proportional distribution across different levels of epilepsy centers

^*^The KDT adoption rate between primary and secondary centers is statistically significant

^#^ The KDT adoption rate between primary and tertiary centers is statistically significant

^**^ The KDT adoption rate between secondary and tertiary centers is statistically significant

**Table 2:** The Patient Group of KDT Served by the Epilepsy Centers

	Pediatric	Adult	Pediatric and adult	Total
Primary centers	18 (90%)	1 (5%)	1 (5%)	20
Secondary centers	51 (67%)	8 (11%)	17 (22%)	76
Tertiary centers	12 (50%)	0 (0%)	12 (50%)	24
Total	81 (68%)	9 (7%)	30 (25%)	120

The numbers in the table represent the number of epilepsy centers, whereas the percentages in parentheses indicate their proportional distribution across different levels of epilepsy centers

With respect to KDT formulation protocols, 10% (12/120) of centers utilized exclusively family prepared food, accounting for 353 total servings. These centers included 15% (3/20) primary, 9% (7/76) secondary, and 8% (2/24) tertiary institutions. Product based KDT were employed by 66% (79/120) of centers, representing 770 total servings, with distributions across 70% (14/20) primary, 75% (57/76) secondary, and 33% (8/24) tertiary centers. 24% (29/120) of centers utilized both preparation methods, accounting for 333 total servings, distributed across 15% (3/20) primary, 16% (12/76) secondary, and 58% (14/24) tertiary centers (see Table 3).

**Table 3:** The KDT Food Used

	Family prepared food	Product based KDT	Both preparation methods	Total
Primary centers	3 (15%)	14 (70%)	3 (15%)	20
Secondary centers	7 (9%)	57 (75%)	12 (16%)	76
Tertiary centers	2 (8%)	8 (33%)	14 (58%)	24
Total	12 (10%)	79 (66%)	29 (24%)	120

The numbers in the table represent the number of epilepsy centers, whereas the percentages in parentheses indicate their proportional distribution across different levels of epilepsy centers

In 2023, the 120 epilepsy centers that implemented KDT reported varying numbers of patients receiving KDT, encompassing all four types of KDT. Among these centers, 77% (92/120) of centers treated 0–10 patients, 12% (14/120) of centers treated 11–20 patients, and 12% (14/120) of centers treated ≥ 20 patients. With respect to specific KDT types, cKDT was offered by 63% (75/120) of the epilepsy centers. Among these centers, 89% (67/75) had been utilizing cKDT since 2013, and 79% (59/75) reported 10 or fewer patients with cKDT in 2023. The MAD was provided by 40% (48/120) of the epilepsy centers, with 90% (43/48) of the centers having implemented the MAD since 2015, and 90% (43/48) of the centers reported 10 or fewer patients on the MAD in 2023. LGIT was offered by 20% (24/120) of the epilepsy centers, 79% (19/24) of which had been using LGIT since 2018, and 79% (19/24) of the centers reported 5 or fewer patients on LGIT. Similarly, the MCT diet was offered by 25% (30/120) of the epilepsy centers, with 77% (23/30) of the centers having adopted the MCT diet since 2020, and 77% (23/30) of the centers reported 5 or fewer patients on the MCT diet (see Table 4).

**Table 4:** The Type of KDT

	cKDT	MAD	LGIT	MCT
Primary centers	9 (45%)	7 (35%)	1 (5%)	4 (20%)
Secondary centers	45 (59%)	26 (34%)	12 (16%)	18 (24%)
Tertiary centers	21 (87%)	15 (62%)	11 (46%)	8 (33%)
Total	75 (63%)	48 (40%)	24 (20%)	30 (25%)

Number of epilepsy centers = 120, number of primary centers = 20, number of secondary centers = 76, number of tertiary centers = 24

The numbers in the table represent the quantity of epilepsy centers, while the percentages in parentheses indicate their proportional distribution across different levels of epilepsy centers

### The efficacy of KDT: perspectives from epilepsy centers

Our survey investigated the spectrum of clinical conditions for which epilepsy centers consider KDT as a potential treatment option. The data provided strong clinical evidence supporting the implementation of KDT, particularly in managing super-refractory status epilepticus (80% adoption rate; 13% primary, 48% secondary, and 81% tertiary institutions) and refractory status epilepticus (77% adoption rate; 12% primary, 47% secondary, and 77% tertiary institutions). Additional conditions with substantial clinical support for KDT included: glucose transporter type 1 deficiency syndrome (70%, 14% primary, 40% secondary, 73% tertiary), infantile spasms (66%, 12% primary, 41% secondary, 54% tertiary), Dravet syndrome (66%, 10% primary, 40% secondary, 65% tertiary), Lennox-Gastaut syndrome (66%, 9% primary, 40% secondary, 73% tertiary), and Ohtahara syndrome (54%, 7% primary, 36% secondary, 46% tertiary).

### The adverse effects of KDT

Safety profile analysis revealed that 74% of epilepsy centers (89/120; comprising 85% primary, 74% secondary, and 67% tertiary centers) reported no adverse effects associated with KDT. Among the 120 epilepsy centers implementing KDT, 27 centers reported adverse gastrointestinal reactions, 14 centers documented hyperlipidemia cases, 12 centers observed hypoglycemia episodes, and 6 centers identified growth/developmental complications. Among the 27 epilepsy centers that reported gastrointestinal adverse reactions, the majority reported ≤ 5 cases during 2023. Among the epilepsy centers that reported adverse effects, only one reported an exceptionally high incidence of hyperlipidemia (30 cases). All other adverse effects occurred at frequencies less than five cases per center throughout the reporting period.

### Implementation challenges and recommendations for KDT adoption

Our investigation revealed several significant barriers to the implementation of KDT, as reported by the participating epilepsy centers. The most prevalent challenge was the limited KDT-related expertise among clinical practitioners, reported by 83% of centers (115/138; comprising 66% primary, 36% secondary, and 4% tertiary centers). This was closely followed by inadequate KDT-related expertise among nutrition specialists, which was cited by 71% of centers (98/138; including 53% primary, 33% secondary, and 4% tertiary centers). Patient-related challenges were also notable, with 56% of centers (78/138; 38% primary, 29% secondary and 8% tertiary centers) reporting low patient acceptance rates due to various factors. Additionally, 56% of the centers (78/138; 50% primary, 20% secondary and 4% tertiary centers) highlighted insufficient physician staffing within the epilepsy center as a significant barrier (see Table 5).

**Table 5:** Barriers to KDT implementation

Barriers	Numbers of centers (percentage)
Limited KDT-specific training among clinical practitioners	115 (83%)
Inadequate KDT-related expertise among nutrition specialists	98 (71%)
Insufficient physician staffing within the epilepsy centers	78 (56%)
Low patient acceptance rates due to various factors	78 (56%)
Concerns regarding potential adverse effects of KDT	49 (36%)
Challenges in family-based KDT meal preparation	33 (24%)
Difficulties maintaining KDT compliance in educational settings	28 (20%)

Number of epilepsy centers = 138

The survey results indicated that 96% of the epilepsy centers (133/138; comprising 100% primary and 92% secondary centers) expressed a need for CAAE support in implementing KDT. Among these centers, the most requested forms of assistance included: specialized clinical training programs on KDT (99%, 132/133; 80% primary and 38% secondary centers), remote expert consultation and telemedicine support (84%, 112/133; 63% primary and 36% secondary centers), development and dissemination of updated KDT clinical guidelines or expert consensus statements (83%, 111/133; 63% primary and 35% secondary centers), and continuing medical education opportunities in KDT management (74%, 99/133; 57% primary and 30% secondary centers) (see Table 6).

**Table 6:** Assistances needed from CAAE to facilitate KDT

Assistances	Numbers of centers (percentage)
Specialized clinical training programs on KDT	132 (99%)
Remote expert consultation and telemedicine support	112 (84%)
Development and dissemination of updated KDT clinical guidelines or expert consensus statements	111 (83%)
Continuing medical education opportunities in KDT management	99 (74%)

Number of epilepsy centers = 133

The epilepsy centers prioritized several key recommendations for the utilization of KDT, with improving patient and family education programs on KDT management emerging as the most crucial recommendation. This was supported by 93% of the respondents (95% primary, 93% secondary, and 88% tertiary epilepsy centers). The second most important recommendation was to strengthen professional training programs for clinicians in epilepsy centers, which received 88% agreement (95% primary, 87% secondary, and 88% tertiary epilepsy centers). Other significant recommendations included expanding the availability of diverse, commercially-available KDT products (83% agreement; 85% primary, 82% secondary, and 83% tertiary epilepsy centers), enhancing KDT-related training for physicians in district and community hospitals (82% agreement; 85% primary, 82% secondary, and 79% tertiary epilepsy centers), and establishing outpatient/remote KDT initiation programs (74% agreement; 70% primary, 74% secondary, and 79% tertiary epilepsy centers) (see Table 7).

**Table 7:** Recommendations to enhance the utilization of KDT

Recommendations	Numbers of centers (percentage)
Improve patient and family education programs on KDT management	111 (93%)
Strengthen professional training programs for clinicians in epilepsy centers	106 (88%)
Expand availability of diverse, commercially available KDT products	99 (83%)
Enhance KDT-related training for physicians in district and community hospitals	98 (82%)
Establish outpatient/remote KDT initiation programs	89 (74%)

Number of epilepsy centers = 120

## Discussion

This survey is the first study in China to investigate the current status of KDT and the barriers to its implementation. Research indicates that 47% of epilepsy centers in China offer KDT and that a limited number of individuals utilize this treatment. According to our survey, approximately half of the epilepsy centers have not adopted KDT, primarily due to a lack of professional knowledge among clinicians and dietitians regarding KDT, as well as difficulties in acceptance by patients and their families for various reasons. The vast majority of epilepsy centers require the following support from the CAAE to implement KDT: providing professional medical training related to KDT, providing regular online guidance from experts, and publishing new KDT guidelines or expert consensus.

### Baseline characteristics of surveyed epilepsy centers

Based on the completed survey responses, our study achieved a response rate comparable to those of previous UK surveys conducted in 2000 (51%) [20] and 2010 (48%) [16]. Moreover, the response rates from epilepsy centers at various levels are quite similar, indicating that our sample is highly representative and can accurately reflect the actual situation across different tiers of epilepsy centers. Approximately half of the epilepsy centers provided services for both adults and children, thereby catering to a broader patient population.

### Clinical application of KDT

Our survey revealed significant variations in the adoption rate of KDT across different tiers of epilepsy centers, with statistically significant differences observed in pairwise comparisons. These disparities may stem from variations in medical resources, technical capabilities, and patient demographics among centers at different levels [19]. Among the centers that implement KDT, the majority primarily treat pediatric patients (aged ≤ 18 years). With respect to the types of KDT formulations, most epilepsy centers prefer using either product based KDT or a combination of product based and family prepared KDT, rather than relying solely on family prepared KDT.

Among the epilepsy centers offering KDT, the cKDT is the most widely implemented, with approximately half of the epilepsy centers providing cKDT, while fewer centers offering MAD, LGIT, and MCT therapy. This phenomenon is similar to the situation in the United Kingdom in the year 2000, where 59% of epilepsy centers utilized cKDT and 40% of centers employed MCT [20]. Recent surveys from both the United Kingdom (2017) and France (2018) reveal a consistent upward trend in KDT adoption among epilepsy centers [15, 18]. In terms of the number of patients using KDT, most epilepsy centers had a relatively small patient population in 2023. This phenomenon is similar to the situation in France in 2018, where 88% of epilepsy centers had 0–20 individuals utilizing KDT annually [18]. Notably, the United Kingdom has seen substantial growth in KDT utilization, with patient numbers increasing from 101 in 2000 to 152 in 2010, and to 754 in 2017 [15]. China's implementation of KDT presents distinct characteristics when compared to other nations, both in terms of the number of centers and patient adoption rates. These differences primarily stem from three key factors: (1) marked regional disparities in healthcare resource allocation, with 2016 data showing approximately 75% of Chinese epilepsy centers concentrated in eastern and western regions [19], This uneven distribution contrasts sharply with the United Kingdom's more geographically balanced network of epilepsy centers [15]; (2) varying sociocultural acceptance of dietary interventions; and (3) economic considerations within China's healthcare system. Comparative analysis of national statistics reveals significant GDP differences between China and the UK, which likely contribute to these observed disparities in KDT implementation and accessibility.

### The efficacy of KDT: perspectives from epilepsy centers

KDT is widely recognized as an established and effective treatment for DRE [21–26] and super-refractory status epilepticus [27, 28]. In clinical practice, epilepsy centers often consider implementing KDT when managing patients with super-refractory or refractory status epilepticus. According to the 2019 Chinese guidelines, KDT has an efficacy rate of 70% in treating these two conditions and has been widely recognized as a conventional therapeutic approach for DRE [29]. To date, only Japan has conducted a nationwide survey specifically focusing on glucose transporter type 1 deficiency syndrome [30]. No country has yet performed a comprehensive investigation regarding other indications for KDT, nor has any country systematically prioritized the clinical applications of KDT across various medical conditions.

### The adverse effects of KDT

Adverse effects, while not always necessitating the discontinuation of KDT, remain a significant concern. Generally, the risk of severe side effects is low, and KDT does not need to be discontinued for most side effects [31]. A considerable proportion of respondents reported no adverse effects. The most frequently reported adverse effect was gastrointestinal reactions, aligning with findings from international research, which suggests that some of these adverse events are temporary and manageable [32]. In a Japanese study with a limited sample size of 39 participants, KDT was associated with a relatively high rate of adverse reactions, with 6 out of 30 patients experiencing adverse effects [30]. The observed discrepancy might be due to the smaller sample size in the Japanese study. Notably, only one center reported 30 cases of hyperlipidemia in 2023, potentially linked to the rapid ketogenesis and high fat intake associated with KDT in this center. Historically, fasting was believed to accelerate ketogenesis. However, recent research has indicated that the gradual initiation of KDT results in a similarly high rate of seizure reduction and a decrease in adverse events [33]. Therefore, it is crucial to minimize the occurrence of adverse events through gradual initiation. Preventing adverse reactions will remain a key priority for CAAE in its future initiatives related to KDT.

### Implementation challenges and recommendations for KDT adoption

As highlighted in the survey, approximately half of the epilepsy centers in China do not implement KDT and require support from the CAAE to overcome existing barriers. Among the key obstacles to KDT utilization, the most significant are the limited KDT-related expertise among clinical practitioners and nutrition specialists. This finding is consistent with a previous study conducted in the United States, which identified a lack of expertise as the most common barrier to adopting KDT as a treatment option [3]. Furthermore, a single-center retrospective observational study emphasized the necessity of educating healthcare professionals about the fundamental principles of dietary therapy [34]. Importantly, research has demonstrated that following the 2009 consensus recommendations, there was a notable increase in referrals for children using KDT between 2009 and 2018 [35]. This underscores the importance of publishing comprehensive guidelines or recommendations on the clinical indications for KDT utilization, as well as detailed protocols for its implementation. Such materials should cover not only the indications, basic principles, common side effects, sample recipes, and monitoring methods [34], but also general patient management in acute settings, including specific guidance on ketonemia monitoring, fasting and refeeding procedures, and drug administration [36]. China released a guideline on KDT in 2019 [29], however, this guideline does not cover the management of patients with acute conditions. In the future, it may be necessary to publish new guidelines to refine and address the various aspects related to KDT. To further promote KDT adoption, enhancing awareness among healthcare providers through national lectures, grand rounds, and continuing education programs is essential. These initiatives increase the knowledge of district and community doctors regarding the indications for KDT, enabling them to better manage patients on a diet who present with unrelated conditions such as colds or other illnesses. By addressing these barriers and providing the necessary resources and training, the implementation and utilization of KDT can be significantly improved.

The implementation of KDT typically involves a multidisciplinary team including physicians, dietitians, patients and their families. Our survey identified low patient acceptance as the third major barrier to KDT adoption, consistent with prior studies [18, 37–39]. Key contributing factors included culture factors, economic constraints, safety concerns related to adverse effects, dietary restrictions and practical difficulties such as meal preparation and cost [39]. These concerns were particularly reflected in the psychological stress experienced by families due to rigid dietary requirements and reduced flexibility [37]. To address these issues, we recommend a comprehensive approach that enhances current practices where most outpatient protocols provide only 2–3 h of KDT training [34]. First, structured education programs should be established, including hands-on training in ketogenic teaching kitchens [40], supplemented with e-health tools for daily management [37, 41] and pre-consultation materials such as brochures, booklets with relevant web addresses and visual aids [34, 37, 41]. Second, follow-up support through phone or email [34], development of more palatable and affordable meal options, and digital tools like carbohydrate monitoring apps should be implemented. This is particularly important since the United Kingdom data showed that poor adherence—rather than treatment ineffectiveness—was the primary reason for discontinuing KDT [20]. Notably, more palatable diet formulations were consistently associated with improved long-term adherence [20]. These combined measures would help overcome existing barriers and improve acceptance and long-term adherence to KDT.

With respect to the implementation of KDT, epilepsy centers require support from the CAAE in several key areas: specialized clinical training programs on KDT, remote expert consultation and telemedicine support, the development and dissemination of updated KDT clinical guidelines or expert consensus statements, and continuing medical education opportunities in KDT management. These findings underscore a gap in physicians' comprehensive understanding of KDT, highlighting the need for targeted educational initiatives. One potential solution is the establishment of a "hub and spoke" model of care, where local centers are connected to a regional pediatric neurology service that serves as an expert resource. This model provides access to state-of-the-art knowledge and management strategies while allowing patients to continue receiving care at their local centers [16]. Additionally, epilepsy centers should be encouraged to adopt KDT by leveraging available resources and training opportunities from established, experienced centers. This collaborative approach would facilitate knowledge transfer, enhance clinical expertise, and ultimately improve the implementation and effectiveness of KDT across a broader network of healthcare providers.

### Limitations

Online surveys offer significant advantages but also have distinct limitations. First, despite a high response rate, some epilepsy centers did not participate in our questionnaire, leading to inevitable gaps or incomplete data. Second, the study focused mainly on the use of KDT and existing barriers, but lacked follow-up data on the long-term clinical outcomes of patients, making it impossible to assess the therapeutic effects comprehensively. Third, our study did not compare the advantages and disadvantages of family-prepared KDT versus product-based KDT. Future research could investigate this aspect to provide practical guidance for patients' dietary choices. Finally, since all the participating centers were members of the CAAE, the findings may not fully reflect the utilization of KDT in grassroots hospitals or settings outside this network. These limitations highlight the need for cautious interpretation of the findings and suggest areas for improvement in future research.

## Conclusions

This survey represents the first large-scale investigation into the implementation of KDT in China. The findings comprehensively reflect the current status of KDT utilization and establish a critical foundation for its future adoption and optimization. To support this effort, the CAAE should prioritize the publication of clinical guidelines, alongside providing professional medical training, refresher courses, and online guidance for healthcare providers. Key barriers to KDT utilization include insufficient KDT-related expertise among physicians and nutritionists. To address these challenges, recommendations include enhancing KDT-related knowledge and education, thereby improving access to this effective treatment for DRE and expanding its availability to a broader patient population.

## Supplementary Information

Supplementary Material 1.

## Data Availability

Not applicable.

## Abbreviations

CAAE: China Association Against Epilepsy
KDT: Ketogenic diet therapy
MAD: Modified Atkins diet
LGIT: Low glycemic index treatment
MCT: Medium-chain triglyceride
