# Supplementary material for: Investigation of the implementation and utilization of ketogenic diet therapy in China: For the Commission on Standardized Development of Epilepsy Centers and Commission on Ketogenic Diet, China Association Against Epilepsy
Source: Acta Epileptol. 2026 Apr 1;8:9. doi: 10.1186/s42494-026-00242-w (PMC13041439; doi:10.1186/s42494-026-00242-w)
Supplement: Supplementary file 1 — Supplementary Material 1. [file 42494_2026_242_MOESM1_ESM.docx]

**Ketogenic Diet Questionnaire**

1.What is the certification level of your epilepsy center by the CAAE?

A Primary epilepsy center (epilepsy clinic)

B Secondary epilepsy center (epilepsy center)

C Tertiary epilepsy center (comprehensive epilepsy center)

2.When was your epilepsy center certified by the CAAE? (please specify the year)

3.Which category of CAAE-accredited epilepsy center does your institution belong to?

A Pediatric epilepsy center

B Adult epilepsy center

C Comprehensive epilepsy center (children and adults)

4.Does your epilepsy center implement the KDT?

A Yes

B No

5.Which patient population is the primary target patient for the KDT program at your center?

A Pediatric patients (Aged ≤ 18 years)

B Adult patients

C Both pediatric and adult patients

6.What KDT preparation methods does your epilepsy center offer to patients?

A Exclusively patient-prepared KDT

B Exclusive formula-based KDT

C Both patient-prepared and formula-based KDT

7.What was the total number of patients who independently prepared their own KDT at your center in 2023?

8.How many patients independently utilized formula-based KDT at your center in 2023?

9.What was the number of patients who employed both preparation methods (patient-prepared and formula-based KDT) at your center in 2023?

10.Which of the following suggestions would facilitate better adoption of the KDT among patients and families?

A Enhance KDT-related training for physicians in district and community hospitals

B Develop comprehensive KDT cookbooks and meal planning guides

C Implement nutritionist‒clinician collaborative care models

D Expand availability of diverse, commercially available KDT products

E Strengthen professional training programs for clinicians in epilepsy centers

F Improve patient and family education programs for KDT management

G Establish outpatient/remote KDT initiation programs

H Other (please specify)

11.What are the primary barriers to implementing KDT in epilepsy centers? (multiple selections allowed)

A Perceived lack of clinical efficacy in epilepsy management

B Insufficient physician staffing within the epilepsy center

C Limited KDT-related expertise among clinical practitioners

D Inadequate KDT-related expertise among nutrition specialists

E Challenges in home-based KDT meal preparation

F Difficulties maintaining KDT compliance in educational settings

G Low patient acceptance rates due to various factors

H Concerns regarding the potential adverse effects of KDT

I Other barriers (please specify)

12. Would your epilepsy center require CAAE support for implementing KDT?

A Yes

B No

13. What types of support would your epilepsy center request from the CAAE for KDT implementation? (Multiple selections allowed)

A Specialized clinical training programs for KDT

B Development and dissemination of updated KDT clinical guidelines or expert consensus statements

C Onsite expert consultation and mentorship programs

D Remote expert consultation and telemedicine support

E Continuing medical education opportunities in KDT management

F Other support services (please specify)

14.What was the total number of patients who received any form of KDT (including all four variants) at your center in 2023?

15.What is the minimum age threshold for initiating KDT at your center?

16.Does your epilepsy center implement cKDT?

A Yes

B No

17.In which calendar year did your center initiate the cKDT?

18.How many patients received cKDT at your center in 2023?

19.Does your epilepsy center implement the MAD?

A Yes

B No

20.In which calendar year did your center initiate the MAD?

21.How many patients received MAD therapy at your center in 2023?

22.Does your epilepsy center implement LGIT?

A Yes

B No

23.In which calendar year did your center initiate the LGIT?

24.How many patients received LGIT therapy at your center in 2023?

25.Does your epilepsy center implement the MCT ?

A Yes

B No

26.In which calendar year did your center initiate the MCT?

27. How many patients received MCT therapy at your center in 2023?

28.Which patient population in your epilepsy center would be considered potential candidates for KD therapy?

1. Refractory status epilepticus
2. Super refractory status epilepticus
3. Tuberous sclerosis complex
4. Pyruvate dehydrogenase deficiency
5. Mitochondrial complex Ⅰ deficiency
6. Ohtahara syndrome
7. Glucose transporter type Ⅰ deficiency syndrome
8. Angelman syndrome
9. Infantile spasm
10. Dravet syndrome
11. Myoclonic-atonic epilepsy
12. Lennox–Gastaut syndrome
13. Febrile infection-related epilepsy syndrome

29.Has your epilepsy center observed any adverse effects associated with the KDT?

A Yes

B No

30 For patients experiencing adverse effects, what are the most frequently reported complications?

A Hyperketonemia

B Hyperlipidemia

C Kidney stones

D Hypoglycemia

E Hypoproteinemia

F Affects growth and development (vitamin deficiency, growth retardation, etc.)

G Gastrointestinal reactions (constipation, diarrhea, etc.)

H Other adverse reactions (please specify)
